# To offer or request? Disclosing variants of uncertain significance in prenatal testing

**DOI:** 10.1111/bioe.12932

**Published:** 2021-08-20

**Authors:** Gabriel Watts, Ainsley J. Newson

**Affiliations:** ^1^ Faculty of Medicine and Health, Sydney School of Public Health, Sydney Health Ethics University of Sydney Sydney Australia

**Keywords:** bioethics, ethical issues, genomics, microarray analysis, prenatal genetic counseling, prenatal screening

## Abstract

The use of genomic testing in pregnancy is increasing, giving rise to questions over how the information that is generated should be offered and returned in clinical practice. While these tests provide important information for prenatal decision‐making, they can also generate information of uncertain significance. This paper critically examines three models for approaching the disclosure of variants of uncertain significance (VUS), which can arise from forms of genomic testing such as prenatal chromosomal microarray analysis (CMA). Contrary to prevailing arguments, we argue that respect for reproductive autonomy does not justify adopting a model on which an offer to disclose VUS is a routine part of genetic counselling. Instead, we contend that a commitment both to solidarity between healthcare providers and pregnant women and to the acceptance of a novel principle of caution under normative uncertainty means that we should instead adopt a model of VUS disclosure that imposes a strong presumption *against* offering to disclose VUS. The upshot of this is that it should be standard practice to only offer to disclose VUS when this is requested by the woman undergoing CMA. We defend our position against claims that arise from an alleged right to such information and that a presumption against an offer will lead to inequity.

## 1 INTRODUCTION

For all the excitement about new genomic technologies, their use in perinatal care has brought new uncertainties, as well as new ethical challenges.^1^ Of particular concern in recent years has been the widespread implementation of chromosomal microarray analysis (CMA) as a tool to aid diagnosis and prognostication during prenatal testing and subsequent decision‐making. CMA is a powerful technology, which has rapidly diffused into prenatal testing settings. It is able to detect sub‐microscopic gains or losses of genetic material on fetal chromosomes, changes that cannot be observed via conventional chromosome analyses.^2^ These changes are known as copy number variants (CNVs) and can be classified in numerous ways, with the aim to indicate whether and how the change might impact the health of the future child.^3^

Because CMA looks at the fetal genome in a finer‐grained way, its use in prenatal testing promises a superior diagnostic yield compared with previous technologies. As a result, it is now a recommended first‐line test for various clinical indications in pregnancy, including the detection of a structural anomaly on ultrasound. It is widely endorsed by professionals and pregnant women.^4^ But CMA also gives rise to ethical tensions, including when it should be used (only after a potential anomaly is identified, or in all pregnancies?), which kinds of findings should be disclosed (only those with clear implications for the health of the child, or broader ones?) and who should decide these matters (health professionals, or pregnant women?).^5^

Ethical tensions regarding the use of prenatal CMA also arise from the identification and potential return of variants of uncertain (or unknown) significance (VUS). A CNV is classified as a VUS when there is not enough information available to say whether it is related to a genetic condition.^6^ An uncertain result may be difficult to incorporate into further decision‐making, particularly given the inherent time pressure in deciding whether to continue or terminate a pregnancy and the paucity of clinical phenotype information to aid interpretation. Pregnant women thus find themselves in a liminal space, where their fetus is deemed neither affected nor healthy.^7^

There is already a substantial literature examining the clinical and psycho‐social consequences of disclosing VUS in the prenatal context. We discuss this further in Section 3.1 below. Still we note here that while pregnant women are generally information‐seekers, there is also broad recognition that VUS disclosure has the potential to increase parental distress^8^ beyond customary pregnancy‐related stresses, including those attributable to ‘lower‐resolution’ forms of prenatal testing.^9^ VUS findings also lead to novel difficulties for genetic counsellors,^10^ for whom the inability to provide unambiguous advice, amongst other things, can become a distinct cause of distress.^11^ Concern has also been expressed that disclosing VUS following CMA can lead to unnecessary terminations of pregnancy.^12^ As the use of CMA (and other technologies to detect CNVs, such as whole‐exome sequencing) in prenatal testing increases, these issues will only become more pressing.

Approaches to offering and disclosing VUS to pregnant women undergoing CMA vary globally—from withholding in the U.K. and Belgium, to routinely reporting them (with prior consent) in the Netherlands and the United States. This may have to do with features of the respective healthcare systems, such as litigation rates, how health provision is funded, and models of decision‐making. But such variation in practice also reflects an ethical dilemma—one underscored by diverse and divergent professional views^13^—regarding whether VUS identified in prenatal CMA should be routinely offered, offered on request, or routinely withheld.

In this paper we critically interrogate an over‐arching question integral to the development and implementation of novel tests in prenatal care: should genetic information about a fetus that has no known clinical significance be withheld, routinely offered, or offered only on request? We analyse this question by considering the strengths and weaknesses of three potential models for disclosing VUS to pregnant women undergoing CMA. In Section 2 we describe and contextualize these models. In Section 3 we reject a model on which disclosure of VUS is routinely withheld from pregnant women. In Sections 4 and 5 we critically compare routinely offering to disclose VUS with providing such results on request. We argue that a model where VUS are disclosed on request—one that imposes a strong presumption *against* routinely offering to disclose VUS—is preferable to one that imposes a strong presumption of universally or routinely offering to disclose VUS. We adopt this position on the grounds that a request model better enacts solidarity with pregnant women and better adheres to a novel principle that we term ‘caution under normative uncertainty’. As such, we conclude that it should be standard practice to only offer to disclose VUS when this is requested by the woman undergoing CMA, and then to do so, or not, in accordance with her considered decision.

## 2 MODELS FOR DISCUSSING VUS WITH PREGNANT WOMEN

The starting point for our analysis is the general recognition that ethical issues posed by the identification (or possible identification) of VUS demand that pregnant women are supported throughout the testing process, such that they both understand the implications of the test, and are ready—as best as they can be—to adapt to whatever results it gives rise to.^14^ Within this over‐arching sphere of support, and mindful of the global variations in practice, we discern three possible models for discussing VUS with pregnant women: a *Withhold Model*, a *Request Model* and a *Routine Offer Model*. Each is described below.

To be clear, none of these models encompass situations where *all* information (both general and specific) regarding possible VUS arising from CMA is *mandatorily withheld* from pregnant women; nor does any model involve information about actual VUS being *mandatorily disclosed*. Such models would not meet a minimum standard of ‘support’ throughout the process of CMA. Instead, each of our three models presumes that the possibility of VUS arising from CMA is always discussed with pregnant women during pre‐test counselling. In post‐test counselling, any VUS findings may or may not be disclosed, in accordance with the ethical considerations held to justify the model.

There is also a question as to whether VUS arising from CMA ought to be included in laboratory reports as a matter of course regardless of whether they will be disclosed to pregnant women, perhaps to aid possible future reinterpretation. We do not address this issue here. Instead, our models are neutral as to whether the treating health professional would have access to these results. They allow, in principle, that information about VUS might be generated but masked prior to reporting (such that the treating health professional does not have access), or that such information is included in laboratory reports (such that they do have access). Our intention, rather, is to step back from the question of how uncertain information can best be disclosed or best be used to inform subsequent decisions (as much of the literature on prenatal CMA has done to date), and consider whether the presumption in perinatal care should be to offer this kind of information, provide it only on request, or withhold it.

With these caveats in place, we consider three basic models for disclosing VUS to pregnant women.

- On a *Withhold Model*, genetic counsellors must not offer to disclose specific actual VUS arising from CMA, and must withhold such information post‐test.
- On a *Request Model* there is a strong presumption against offering to disclose VUS post‐test. However, if such information is requested by the pregnant woman, then genetic counsellors should (after discussion) disclose information regarding VUS in accordance with the pregnant woman s considered decision. Here a request for VUS disclosure may be interpreted broadly, so as to include, for instance, a request to know ‘all available information'; a pregnant woman does not need to request VUS by name.
- On a *Routine Offer Model*, there is a strong presumption in favour of genetic counsellors offering to disclose VUS. On this model it is standard or routine practice to offer to disclose VUS to the pregnant woman, and to disclose or not disclose VUS in accordance with her considered decision.

Here we intend a ‘strong presumption’ either for or against disclosing VUS to have at least the force of a ‘should’ and at most the force of a ‘must’. This is to allow for a variety of particular instantiations of these basic models, where an instance of a *Routine Offer Model* is characterized by an offer to disclose VUS, and an instance of a *Request Model* is characterized by abstaining from making such an offer. In contrast, an instance of a *Withhold Model* is characterized by a strict injunction both against making such an offer and against disclosing VUS post‐test.

## 3 AGAINST THE *WITHHOLD MODEL*

As mentioned above, disclosing VUS in prenatal CMA has the potential to increase distress and anxiety beyond customary pregnancy‐related stresses. This occurs against the well‐observed phenomenon of pregnant women seeking certainty and reassurance from prenatal testing, as well as seeking all available information from such tests as a means to provide such reassurance.^15^ However, pregnant women can be ‘blindsided’ by the results, experiencing shock and worry and describing the information as ‘toxic knowledge’.^16^ Receiving a VUS can place pregnant women in a liminal ‘therapeutic gap’, one in which there is a technological ability to do CMA but no ability to fully understand the results.^17^ This gap both causes worry and frustration and generates a ‘dilemma of action’, because there is no clear path to confidently make a decision in light of the information received.^18^ As such, many pregnant women embark upon ‘exhausting and potentially fruitless information‐gathering in attempts to assert certainty’.^19^ Moreover, even after the birth of a ‘seemingly typical’ child, parents can continue to be anxious, and in extreme situations see their children as ‘patients in waiting’, accessing early‐intervention therapies and engaging in other medical monitoring even though their child is well and meeting all developmental milestones.^20^ Parents who receive VUS have also reported less satisfaction and greater regret over their decision to have CMA testing in comparison with those who did not.^21^

The distress arising from VUS disclosure is not limited to expectant parents—genetic counsellors are impacted too. Bernhardt et al., for example, found that over a third of genetic counsellors would not feel comfortable undertaking such counselling, and just over 40% would feel at ease in helping a woman to decide about a pregnancy termination following disclosure of a VUS.^22^

In light of such concerns, ought we to adopt a *Withhold Model*, on which genetic counsellors must not offer to disclose specific actual VUS arising from CMA, and must withhold such information post‐test? We believe the bar for routinely precluding the disclosure of VUS is a high one. For instance, we do not think that the chance of harm arising from distress—whether to expectant parents or to genetic counsellors—provides a compelling reason to adopt a *Withhold Model*. For while it is true that the uncertain significance of VUS means that disclosing them carries an elevated risk of distress, it is also true that (a) uncertainty is an ineradicable feature of reproductive decision‐making generally and prenatal testing specifically, and (b) responses to genomic uncertainty will vary widely amongst individuals.^23^ Indeed, in some of the studies noted above, parents did go on to rationalize and adapt to the uncertainty, or were ultimately glad to have this information.^24^ We also note that those who raise concern about harms from VUS do not advocate for a withhold model.^25^ This being the case, rather than always withholding VUS, we ought to attempt to pre‐empt and manage any distress arising from their disclosure in those instances in which it is sought^26^ yet also potentially harmful.

There is, however, a stronger argument than potential distress for adopting a *Withhold Model* of VUS disclosure. This is that the uncertain significance of VUS entails that they cannot bear materially on the question of apparent or suspected fetal anomalies, and therefore should not be disclosed on grounds of their irrelevance.^27^ We can couple this argument with the observation that pregnant women might feel obligated to know all available information about their fetus, regardless of its relevance, on the supposition that ‘the more information the better’.^28^ Thus, if we do not adopt a *Withhold Model*, we risk creating situations where women choose to have VUS disclosed to them not because they want to know such information (perhaps they believe that knowledge of VUS cannot bear materially on the question of apparent or suspected fetal anomalies, and do not want the added uncertainty) but because they feel obligated to know all available information.

As we make clear in Section 5.1, we believe that the obligation to know all available information about a fetus on the grounds of ‘the more information the better’ is problematic. However, at the same time we do not believe that guarding against such feelings of obligation provides sufficient reason to impose a universal ban on disclosing VUS. Our reason for this is that it is impossible to determine in advance what people will find relevant to reproductive decision‐making, and that, all else being equal, we can allow pregnant women access to information that could materially affect their subsequent decisions.^29^ It is conceivable, for instance, that obtaining a VUS result may provide insight into a pregnant woman's level of comfort with the very possibility of fetal anomalies, and therefore that such a disclosure is at least potentially material to her.^30^ To be clear, we do not see such oblique benefits of VUS disclosure as always or necessarily outweighing its potential harms.^31^ Rather, our point is that without being able to place any *a priori* restrictions on relevance, we *ought not* to veto VUS disclosure. We ought, rather, to mitigate against any problematic feelings of obligation to have VUS disclosed without resorting to a strict injunction against providing information that could potentially be material to reproductive decision‐making.

We allow that there may be cases in which, all things considered, withholding the reporting of VUS is justified.^32^ We see good reason to withhold the reporting of this information about a fetus were such information desired solely out of mere interest in knowing the information, rather than to assist with reproductive decision‐making. Yet, at least as things stand, this is not generally the case with the reporting of VUS after CMA. Thus we do not believe there is sufficient reason to make a *Withhold Model* the standard practice when counselling women undergoing CMA, and so we conclude that we ought not adopt such a model.

## 4 A *ROUTINE OFFER MODEL* OR A *REQUEST MODEL*?

If we ought not adopt a *Withhold Model*, how then should information about VUS be made available to pregnant women? There are two remaining models: a *Routine Offer Model*, and a *Request Model*. Of these, the *Routine Offer Model* has received the most attention (and endorsement from pregnant women^33^), so we will begin with it.

### 4.1 An existing argument for adopting a *Routine Offer Model*

Adopting a *Routine Offer Model* can take several forms. For example, Halliday et al.^34^ have trialled a system where pregnant women are offered a choice between ‘extended’ reporting—including VUS—and ‘targeted’ reporting—in which VUS are not reported. These researchers recommend adopting this choice‐based system as standard clinical practice on the ground that, all else being equal, it ‘seems imperative to involve expectant couples in their own reproductive decision‐making which will impact on the rest of their lives’.^35^ This position echoes that of McGillivray et al. (among others),^36^ who argue that ‘the autonomy of women and their right to information from high‐resolution genome‐wide CMA’^37^ justifies routinizing an offer to disclose VUS, because ‘a woman's autonomy in the context of informed consent is the most important ethical consideration’.^38^

Insofar as such claims appeal to respect for reproductive autonomy they have significant normative weight. This said, there is a need to tread carefully. For one, we must be careful not to equate respect for reproductive autonomy with respect for informed reproductive choices (whether in the context of informed consent or otherwise). For while autonomous reproductive choices arguably are informed ones,^39^ it is now well‐recognized that if respecting a person's autonomy is merely respecting their informed choices, whatever those may be (in this case whether to have VUS disclosed), then we need a further argument as to why we ought not to respect *all* their choices, no matter how uninformed, or irrational.^40^

Instead, as we understand it, respect for a woman's reproductive autonomy requires genetic counsellors to actively facilitate choices that conform to norms of deliberative reflection. We regard such norms as being grounded, minimally, in a person's being appropriately responsive to reasons for making a particular decision, given their values.^41^ Here we understand ‘actively facilitating’ such choices to be a less demanding requirement than that of seeking to ‘ensure’ that a person's choices conform to norms of deliberative reflection; this is necessary, we believe, in order to retain a notion of autonomous choice within a co‐operative decision‐making environment.^42^ Within this environment, ‘norms of deliberative reflection’ provide an ideal at which to aim when facilitating reproductive decisions.^43^ The demand, then, is to support women to make decisions that are consistent with their values (and any other relevant values), through the provision of adequate opportunities to deliberate and reflect upon their decisions.^44^

This being the case, it will not be enough for any *Routine Offer Model* to merely provide pregnant women with the information necessary for them to make an informed choice whether to have VUS disclosed.^45^ Rather, it is only if by adopting such a model we actively facilitate reproductive decisions that conform to norms of deliberative reflection that our doing so can be said to truly respect the reproductive autonomy of pregnant women. Towards this point, McGillivray et al. say that the goal of securing informed reproductive choices through a routine offer to disclose VUS ‘will only be realised if women have access to clinicians skilled in pre‐test and post‐test genetic counselling to inform and assist their decision making’.^46^ We agree. However, the reason we agree is because access to skilled genetic counselling actively facilitates decisions that conform to norms of deliberative reflection, not because respect for reproductive autonomy demands that we adopt a *Routine Offer Model*. Rather, respect for reproductive autonomy demands that we adopt whichever model is the most appropriate means of facilitating reproductive decisions that conform to norms of deliberative reflection.

### 4.2 Routinization and norms of deliberative reflection

The key question, then, is whether adopting a *Routine Offer Model* is the most appropriate means for genetic counsellors to facilitate reproductive decisions that conform to norms of deliberative reflection. Here, given that the crucial difference between a *Routine Offer Model* and a *Request Model* is that in the former case the offer to disclose VUS is routinized, while in the latter case it is standard practice to abstain from offering to *disclose* VUS, the key aspect to consider is the effect of the offer's routinization upon the ability of pregnant women to make decisions that conform to norms of deliberative reflection.

Concerns about the detrimental impact of routinization upon the capacity for autonomous decision‐making are well documented, especially in the context of antenatal screening.^47^ A typical worry is that routine healthcare procedures have the potential to become inadvertently directive, because the fact that the procedure is part of a standard offer from a healthcare provider will carry an implicit presumption that it should be accepted.^48^ Another more specific worry is that adopting a *Routine Offer Model* could serve to orient discussion of VUS towards the offering of a choice to have VUS disclosed or not, rather than towards the eliciting of considered values regarding reproductive decision‐making.

One response to concerns regarding routinization is to hold that its potentially negative impacts can be mitigated though ‘[a]ttention to counselling and information provision’.^49^ In the context of VUS, one could argue that concerns over the implicit directivity of a *Routine Offer Model* can be mitigated through the design of the offer and the use of tools such as decision aids.^50^ Likewise, one could claim that it is possible to offset the concern that routinizing the offer to disclose VUS will skew discussions away from the eliciting of considered values (and towards merely offering a choice) by establishing practice guidelines (or revising existing guidelines) that stress the importance of eliciting considered values.

This said, however, it does not follow that routinizing the offer of VUS disclosure is the most appropriate means of respecting reproductive autonomy. Indeed, even if it turns out that, as a matter of fact, routinization does not interfere with reproductive autonomy,^51^ this does not provide a reason to prefer a *Routine Offer Model* of VUS disclosure to a *Request Model*. Rather, what is needed is an argument that routinizing the offer of VUS disclosure better facilitates reproductive decisions that conform to norms of deliberative reflection, as opposed to not routinizing such an offer.

Here it could be argued that the *Routine Offer Model* reduces the likelihood that the discussion of VUS is either intentionally or negligently omitted during pre‐test counselling. Yet, by the same measure, while this is a foreseeable positive effect of the *Routine Offer Model*, it is not obvious that routinization is a better means of safeguarding such omissions than clear practice guidelines that outline how and why the possibility of VUS ought to be discussed pre‐test. As it stands, we see no obvious benefit in adopting a *Routine Offer Model* over a *Request Model* as a means of respecting reproductive autonomy.

### 4.3 Can we otherwise justify adopting a *Routine Offer Model*?

So far, we have argued that respect for reproductive autonomy does not justify adopting a *Routine Offer Model* over a *Request Model*. However, we also need to consider whether there are other grounds to support adopting a *Routine Offer Model*. There are two such prima facie grounds for preferring a *Routine Offer Model* to a *Request Model*: the purported right to personal information, and equitable access to healthcare information.

#### 4.3.1 A right to personal information?

McGillivray et al. claim that a woman's ‘right to information from high‐resolution genome‐wide CMA’^52^ is a ground for not intentionally withholding VUS disclosure. Assuming that there is such a ‘right to information’, we agree. It is a different question, however, whether any right to personal information justifies adopting a *Routine Offer Model* of VUS disclosure over a *Request Model*. One thought here is that adopting a *Routine Offer Model* universalizes access to information regarding VUS, whereas a *Request Model* effectively limits access to such information to those who ask for it.^53^ Thus if a right to personal information is an important ground for acting in reproductive ethics, it follows that we ought to adopt a *Routine Offer Model*.

However, it is open to debate whether a right to personal information is of special ethical importance, independent of respect for reproductive autonomy. For the purported value of such a right, as distinct from the role that access to information plays in facilitating autonomous decision‐making, is surely connected to the role that access to information plays in the generation of *knowledge*. Yet in the case of VUS, it is not at all obvious that universalizing access to VUS reporting will generate significant amounts of new knowledge.^54^ We argued in Section 3 above that such new knowledge may be relevant, however minimally, to a women's reproductive decision‐making, and thus we ought not to adopt a *Withhold Model*. Yet, considered apart from its relevance to reproductive decision‐making, that is, simply in terms of the knowledge that is produced, we do not think that VUS disclosure generates enough new knowledge to warrant preferring *Routine Offer Model* to a *Request Model* on the ground of a purported right to personal information, because of the very tenuous chance that this information will generate knowledge. This is especially so given that a *Request Model* does not preclude discussing the possibility of VUS, nor disclosing actual VUS if this is requested.

#### 4.3.2 Equitable access to healthcare information

A stronger ground for preferring a *Routine Offer Model* to a *Request Model* is that equitable access to healthcare information is of independent value in reproductive ethics. Here it could be argued that a *Request Model*, insofar as it places the onus upon pregnant women to ask for VUS disclosure, effectively limits such information to persons with higher health literacy or higher confidence to raise the topic with their genetic counsellor. This then creates a disparity of access to healthcare information. Adopting a *Routine Offer Model*, in contrast, ensures equitable access to healthcare information because everyone is offered disclosure of VUS.

While equitable access to healthcare information is certainly an important ground for acting in reproductive ethics, it does not follow that adopting a *Routine Offer Model* is the appropriate means of addressing disparities in health literacy when it comes to reproductive decision‐making. For it is arguably not the routinization of an offer to disclose VUS that ensures equitable access to healthcare information, but the equitable provision of quality pre‐ and post‐test counselling. Indeed, while it is necessary to ensure that women have access to quality pre‐ and post‐test counselling so as to facilitate reproductive decisions that conform to norms of deliberative reflection, the provision of skilled genetic counsellors to assist with a pregnant woman's reproductive decision‐making is also mandated by a concern for equitable access to relevant and good‐quality healthcare information and support. This requirement will not be met simply by a routine offer of uncertain information. As such, there is no reason to think that adopting a *Routine Offer Model* is more equitable than a well‐implemented *Request Model*, insofar as both involve the provision of skilled genetic counselling to support decision‐making. Ultimately then, we conclude that issues of equitable access do not provide a compelling reason to prefer a *Routine Offer Model* to a *Request Model*.

## 5 AN ARGUMENT FOR ADOPTING A *REQUEST MODEL*

We have argued above that, as things stand, there is no compelling reason to prefer a *Routine Offer Model* of VUS disclosure to a *Request Model*. Instead we have claimed that a well‐implemented *Request Model* is at least as appropriate as a *Routine Offer Model* as concerns respect for reproductive autonomy, a right to personal information, and equitable access to healthcare information. However, we have not yet provided any reason to prefer a *Request Model* of VUS disclosure over a *Routine Offer Model*. We will conclude, then, by giving two reasons for preferring a *Request Model* over a *Routine Offer Model*. The first is solidarity between healthcare providers and pregnant women, and the second is a novel principle that we call ‘caution under normative uncertainty’.^55^

### 5.1 Solidarity

A number of authors have stressed the importance of solidarity as a ground for acting in bioethics.^56^ Debates as to the nature of solidarity are ongoing, yet there is agreement that our ‘rising’ on behalf of, or ‘standing’ beside, the vulnerable is of fundamental importance when translating general ethical principles into concrete actions.^57^ In light of what we have argued above, we think that the disclosure of information regarding VUS to pregnant women is an exemplary instance of an ethical dilemma in which heavily cited grounds for action—notably reproductive autonomy—are insufficiently action‐guiding. As such, we believe that demands for healthcare providers to stand in solidarity with pregnant women undergoing CMA ought to be accorded significant normative weight in deliberations regarding the disclosure of VUS.

To this end, it is useful to consider the issue of VUS disclosure through the lens of what Hashiloni‐Dolev et al. have labelled ‘Pandora's Pregnancy’.^58^ They argue that prenatal tests like CMA ‘mark a watershed of change fuelled by growing commercialisation and abundance of information (often in the form of probabilities and chance)’.^59^ More precisely, they argue that the increasing use of these technologies has seen that ‘the sociopsychological burden of uncertainty is shifted to patients, with physicians (as well as human geneticists and genetic counsellors) performing “bridging work” instead of managing users' or patients' complaints/expectations’.^60^ In this environment, to become pregnant is to open a Pandora's box of complex and uncertain information. And, as Werner‐Lin et al. note, ‘…if parents had had a better appreciation of what they were in for, at least some of them would have declined to enrol in CMA testing or consent to learn VUS data’.^61^

We do not wish to take a position as to the accuracy of such claims regarding the work of genetic counsellors. However, we do want to suggest that the notion that burdens of uncertainty or regret ought not to fall entirely on pregnant women speaks to an underlying recognition that any such burdens ought to be held in common. Here the problem is not, or is not simply, that shifting these burdens onto pregnant women undermines their reproductive autonomy. Rather, there has been a breakdown in solidarity between health professionals and pregnant women, such that their joint struggle becomes a lone stand. In this way, solidarity in the face of uncertainty and potential regret is revealed to be a fundamental ethical dimension of the relationship between pregnant women and genetic counsellors.

If this is the case, however, then what does acting in solidarity with pregnant women demand? Here we suggest that solidarity between genetic counsellors and pregnant women imposes a strong presumption against the provision of information for information's sake. We expect this to be a controversial point. For, as noted already, a number of studies have found that women opt to have VUS disclosed to them on the grounds that ‘the more information the better’. However, as we see it, this notion is a product of the commercial and other drivers of testing that Hashiloni‐Dolev et al. describe,^62^ or what Werner‐Lin et al. refer to as the ‘almost magical’ belief in the power of genetic testing.^63^ Seeking information has become so routine that a test offer almost carries with it an expectation that the offer will be accepted, and the desire for information can outweigh concern about what the quality or certainty of the information might be, or what decisions it might enable.^64^ This also includes a technological imperative to utilize novel genomic technologies on the grounds of their mere availability, rather than their expected benefits: an imperative that exists in both user‐pays and state‐funded healthcare environments.^65^ Indeed, the various ethical dilemmas generated by VUS are as much a symptom of technological imperatives in healthcare as they are a product of the increasing diagnostic uncertainty produced by technological advances, and it is legitimate to resist their demands.

This being the case, we regard solidarity between healthcare providers and pregnant women as providing a reason to prefer a *Request Model* over a *Routine Offer Model*. More precisely, we believe that such solidarity demands we adopt the model of VUS reporting that is least likely to promote the decision to receive information about VUS on the ground that ‘the more information the better’. And here, since adopting a *Request Model* imposes a strong presumption against offering to disclose VUS, whereas adopting a *Routine Offer Model* imposes a strong presumption in favour of offering to disclose VUS, we believe that solidarity between healthcare providers and pregnant women justifies adopting a *Request Model* rather than a *Routine Offer Model*.

### 5.2 Caution under normative uncertainty

Alongside the diagnostic uncertainties that VUS engender, we have seen that their disclosure to pregnant women is also plagued by normative uncertainty as to which ethical considerations ought to form the basis of our actions. This is to say that even if we were to know, or agree upon, the consequences of adopting a *Routine Offer Model* of VUS disclosure over a *Request Model*, or vice versa—for example that doing so would interfere with reproductive autonomy, or would not—we would nevertheless be unsure as to what we ought to do. For, as we have shown, there are a number of ethical considerations in play—equitable access to healthcare, access to information, respect for reproductive autonomy and solidarity. These must be weighed both alongside and against each other, and agreeing on the non‐moral facts of the matter does not necessarily give us insight into how we should balance these moral concerns.

The notion of normative uncertainty has recently been used to help frame decision‐making principles in the face of intractable ethical disputes between competing moral theories.^66^ We contend, however, that the same considerations can also apply to novel ethical dilemmas that may well be resolvable, but where, given the novelty of the situation that prompts the dilemma, it is presently unclear what the most important ethical consideration is.^67^ The thought here is that while disputes between competing ethical theories may well be timeless, ethical thinking occurs in time, and normative uncertainty can arise from tractable moral ignorance as well as from intractable theoretical conflicts.

Of course, concerns about normative uncertainty do not mean that we cannot make any recommendations as to how people ought to act. Pregnant women and genetic counsellors are perfectly capable of acting under uncertainty, both diagnostic and normative, and so long as we supply reasons for adopting a *Request Model* over a *Routine Offer Model* then we are justified in doing so. Our point, rather, is that the existence of normative uncertainty over which model of VUS reporting we ought to adopt is *itself* a reason to prefer a *Request Model* to a *Routine Offer Model*.

More precisely, we can appeal to a meta‐normative principle governing actions under normative uncertainty which we call ‘caution under normative uncertainty’. This principle holds that in cases of normative uncertainty, abstaining from acting is preferable to acting: for if we presume that moral understanding improves over time, then we will be better placed in the future to decide what to do than we are now.^68^ In this particular instance, we can say that abstaining from making an offer to disclose VUS is preferable to routinely doing so, at least as things presently stand, and therefore that adopting a *Request Model* is preferable to adopting a *Routine Offer Model*.

This meta‐normative principle is a close cousin of the ‘precautionary principle’.^69^ However, whereas the ‘precautionary principle’ is a *first‐order* normative principle that is justified on the ground that the potential harms of certain actions outweigh any benefits, thus mandating caution, ‘caution under normative uncertainty’ is a *second‐order* normative principle that is justified on the ground that moral understanding improves over time. As such, the principle of ‘caution under normative uncertainty’ is sensitive to temporal pressures to make a decision in a way that the ‘precautionary principle’ is not. For if the need to decide becomes pressing, such that there is little time for moral understanding to improve, then our current moral knowledge is the best we can hope for and provides a compelling reason for the decision made. As such, it may well be that ‘caution under normative uncertainty’ justifies taking moral risks that the ‘precautionary principle’ precludes—but only when such risks have independent first‐order moral justification, and there is little time to improve one's understanding of what to do.^70^

It could happen, for instance, that developments in technology or knowledge render our adopting a *Routine Offer Model* a pressing matter of social justice, or even of upholding a right to access personal information. Were this to happen then the principle of ‘caution under normative uncertainty’ would provide us with a reason to prefer a *Routine Offer Model* over a *Request Model*. However, in light of what we have argued above, we do not think that this is currently the case. Thus we conclude that ‘caution under normative uncertainty’ provides further grounds for adopting a *Request Model* of VUS disclosure, at least as things presently stand.

## 6 CONCLUSION

Pregnancy is inherently uncertain. The disclosure of VUS arising from CMA often introduces an extra, and potentially distressing, form of uncertainty—placing pregnant women in a liminal space where their fetus is neither healthy nor affected with a genetic condition. To date, much of the ethical and clinical debate over disclosing VUS has considered how to manage the uncertainty such disclosure generates. Our paper has reframed this debate to consider whether VUS disclosure ought to be allowed at all, and if so, what model of VUS disclosure we ought to adopt.

We have argued against universally withholding VUS from pregnant women who request this information (when doing so is supported by quality pre‐ and post‐test counselling). Instead some form of VUS disclosure ought to be allowed. Yet, even so, we do not think that respect for reproductive autonomy justifies adopting a model of VUS disclosure in which an offer to disclose VUS is a routine part of genetic counselling. Instead we contend that solidarity between healthcare providers and pregnant women demands that we adopt the model of VUS disclosure that is least likely to encourage the seeking and provision of information for information's sake. This, we argue, justifies adopting a *Request Model* of VUS disclosure over a *Routine Offer Model*.

At the same time, we recognize that the ethical dilemmas posed by disclosing VUS are fraught with normative uncertainty. And while we believe that there are good grounds for preferring a *Request Model* to a *Routine Offer Model*, we remain fully cognisant of the difficulties involved. As such, contrary to prevailing arguments—which have promoted reproductive autonomy as being of fundamental importance—we have refrained from making such trumping claims regarding what the most important ethical considerations might be. Furthermore, we have argued that this normative uncertainty is a further reason to prefer a *Request Model* to a *Routine Offer Model*. For there is nothing wrong with approaching normative uncertainty with caution, and adopting a *Request* model for disclosing VUS. Indeed, on the hopeful assumption that advances in moral knowledge will give us a clearer idea of which model of VUS disclosure we ought to adopt, abstaining from routinely offering to disclose VUS is the right thing to do.

## Footnotes

1 Werner‐Lin, A., McCoyd, J. L. M., & Bernhardt, B. A. (2019). Actions and uncertainty: How prenatally diagnosed variants of uncertain significance become actionable. *Hastings Center Report, 49*(3), S61–S71.

2 Levy, B., & Wapner, R. (2018). Prenatal diagnosis by chromosomal microarray analysis. *Fertility and Sterility, 109*(2), 201–212.

3 Variant classifications include: benign, pathogenic (clinically significant), susceptibility loci (a pathogenic change that has variable clinical presentation) or as a variant of uncertain (or unknown) significance. See, for example, Muys, J., Blaumeiser, B., Janssens, K., Loobuyck, P., & Jacquemyn, Y. (2020). Chromosomal microarray analysis in prenatal diagnosis: Ethical considerations of the Belgian approach. *Journal of Medical Ethics, 46*, 104–109.

4 While we recognize that not all pregnant individuals will be women, we use the word ‘woman’ in this paper. This may denote either the pregnant person themselves, or in conjunction with a partner. See, for example, Evans, M. I., Wapner, R. J., & Berkowitz, R. L. (2016). Noninvasive prenatal screening or advanced diagnostic testing: Caveat emptor. *American Journal of Obstetrics and Gynecology, 215*(3), 298–305; van der Steen, S. L., Diderich, K. E. M., Riedijk, S. R., Verhagen‐Visser, J., Govaerts, L. C. P., Joosten, M., … Galjaard, R. J. H. (2015). Pregnant couples at increased risk for common aneuploidies choose maximal information from invasive genetic testing. *Clinical Genetics, 88*(1), 25–31.

5 See, for example, Shkedi‐Rafid, S., Fenwick, A., Dheensa, S., Wellesley, D., & Lucassen, A. M. (2016). What results to disclose, when, and who decides? Healthcare professionals' views on prenatal chromosomal microarray analysis. *Prenatal Diagnosis, 36*, 252–259; de Jong, A., Dondorp, W. J., Macville, M. V., de Die‐Smulders, C. E., van Lith, J. M., & de Wert, G. M. (2014). Microarrays as a diagnostic tool in prenatal screening strategies: Ethical reflection. *Human Genetics, 133*(2), 163–172; Stark, Z., Gillam, L., Walker, S. P., & McGillivray, G. (2013). Ethical controversies in prenatal microarray. *Current Opinion in Obstetrics and Gynecology, 25*, 133–137; Rosenfeld, J. A., McKinlay, R. J., & Gillam, L. H. (2012). Genetic counselling and ethical issues with chromosome microarray analysis in prenatal testing. *Prenatal Diagnosis, 32*(4), 389–395; Shuster, E. (2007). Microarray genetic screening: A prenatal roadblock for life? *Lancet, 369*, 526–529.

6 It is estimated that a VUS is identified in around 6% of pregnancies that have a structural anomaly detected on ultrasound; and in 1%–2% of pregnancies where no such anomaly is present: Levy & Wapner, op. cit. note 2; Evans et al., op. cit. note 5.

7 Lou, S., Petersen, O. B., Lomborg, K., & Vogel, I. (2020). How do geneticists and prospective parents interpret and negotiate an uncertain prenatal genetic result? An analysis of clinical interactions. *Journal of Genetic Counselling, 29*, 1221–1233.

8 Encompassing maternal distress, partner distress, couple distress or family distress—in that concerns about distress could apply to any person involved in the pregnancy (e.g., known gamete donors, women acting as surrogates, etc.).

9 Werner‐Lin et al., op. cit. note 1; Desai, P., Haber, H., Bulafka, J., Russell, A., Clifton, R., Zachary, J., … Chung, W. K. (2018). Impacts of variants of uncertain significance on parental perceptions of children after prenatal chromosome microarray testing. *Prenatal Diagnosis, 38*, 740–747; Lou, S., Lomborg, K., Lewis, C., Riedijk, S., Petersen, O. B., & Vogel, I. (2020). ‘It's probably nothing, but…’ Couples' experiences of pregnancy following an uncertain prenatal genetic result. *Acta Obstetricia et Gynecologica Scandinavica, 99*(6), 791–801; Harding, E., Hammond, J., Chitty, L. S., Hill, M., & Lewis, C. (2020). Couples experiences of receiving uncertain results following prenatal microarray or exome sequencing: A mixed‐methods systematic review. *Prenatal Diagnosis, 40*, 1028–1039; Bernhardt, B. A., Soucier, D., Hanson, K., Savage, M. S., Jackson, L., & Wapner, R. J. (2013). Women's experiences receiving abnormal prenatal chromosomal microarray testing results. *Genetics in Medicine, 15*(2), 139–145.

10 Hereinafter, we use ‘counsellor’ to denote any health professional who is facilitating pre‐ and post‐test discussion about CMA with a pregnant woman.

11 Richardson, A., & Ormond, K. E. (2018). Ethical considerations in prenatal testing: Genomic testing and medical uncertainty. *Seminars in Fetal & Neonatal Medicine, 23*(1), 1–6; Bernhardt, B. A., Kellom, K., Barbarese, A., Faucett, W. A., & Wapner, R. J. (2014). An exploration of genetic counselors' needs and experiences with prenatal chromosomal microarray testing. *Journal of Genetic Counselling, 23*(6), 938–947.

12 Vetro, A., Bouman, K., Hastings, R., McMullan, D. J., Vermeesch, J. R., Miller, K., … van Ravenswaaij‐Arts, C. M. A. (2012). The introduction of arrays in prenatal diagnosis: A special challenge. *Human Mutatation, 33*, 923–929.

13 See, for example, Werner‐Lin et al., op. cit. note 1; Evans et al., op. cit. note 5; Shkedi‐Rafid et al., op. cit. note 6.

14 See, for example, Werner‐Lin, A., Walser, S., Barg, F. K., & Bernhardt, B. A. (2017). ‘They can't find anything wrong with him, yet’: Mothers' experiences of parenting an infant with a prenatally diagnosed copy number variant (CNV). *American Journal of Medical Genetics A, 173*(2), 444–451; Levy & Wapner, op. cit. note 1; Werner‐Lin et al., op. cit. note 1; Desai et al., op. cit. note 10; Richardson & Ormond, op. cit. note 12.

15 Baker, J., Shuman, C., Chitayat, D., Wasim, S., Okun, N., Keunen, J., … Silver, R. (2018). Informed decision‐making in the context of prenatal chromosomal microarray. *Journal of Genetic Counselling, 27*(5), 1130–1147; Oneda, B., & Rauch, A. (2017). Microarrays in prenatal diagnosis. *Best Practice & Research: Clinical Obstetrics & Gynaecology, 42*, 53–63; van der Steen et al., op. cit. note 5; Lou et al., op. cit. note 8; Richardson & Ormond, op. cit. note 12; Harding et al., op. cit. note 10; Bernhardt et al., op. cit. note 10. Bernhardt et al. state that pregnant women see such information as ‘too good to pass up’ and that there is no reason to decline it.

16 Bernhardt et al., op. cit. note 10; Harding et al., op. cit. note 10.

17 Werner‐Lin et al., op. cit. note 1.

18 Werner‐Lin et al., op. cit. note 1; Harding et al., op. cit. note 10.

19 Werner‐Lin et. al., op. cit. note 1, p. S63; see also Lou et al., op. cit. note 10.

20 Bernhardt et al., op. cit. note 10; Werner‐Lin et al., op. cit. note 15; Lou et al., op. cit. note 10.

21 Desai et al., op. cit. note 10.

22 Bernhardt et. al., op. cit. note 12.

23 See Newson, A. J., Leonard, S. J., Hall, A., & Gaff. C. L. (2016). Known unknowns: Building an ethics of uncertainty into genomic medicine. *BMC Medical Genomics, 9*(57), 1–8; Lou et al., op. cit. note 8; Werner‐Lin et al., op. cit. note 1; Richardson & Ormond, op. cit. note 12.

24 Rubel, M. A., Werner‐Lin, A., Barg, F. K., & Bernhardt, B. A. (2017). Expert knowledge influences decision‐making for couples receiving positive prenatal chromosomal microarray testing results. *Culture, Medicine and Psychiatry, 41*, 382–406; Desai et al., op. cit. note 10; Lou et al., op. cit. note 10.

25 Werner Lin et al., op. cit. note 1.

26 Per our qualification to the *Request Model* above, here ‘sought’ could be interpreted broadly to mean instances where a genetic counsellor determines that a pregnant woman is in effect seeking this information, whether she has in fact articulated it in precise terms.

27 Hofmann, B. (2016). Incidental findings of uncertain significance: To know or not to know—that is not the question. *BMC Medical Ethics, 17*(13), 1–9, p. 6. This claim, of course, relates to the objective relevance of any VUS result. We note and accept that the literature on the relevance of VUS is more nuanced than this, in that there is at times a *perception* that VUS are relevant or VUS information is used in other non‐medical ways—we return to this latter point briefly below.

28 We thank an anonymous reviewer for this observation.

29 Although see Section 4.3.1 below for a clarification as to why this is.

30 McGillivray, G., Rosenfeld, J. A., McKinlay, R. J., & Gillam, L. H. (2012). Genetic counselling and ethical issues with chromosome microarray analysis in prenatal testing. *Prenatal Diagnosis, 32*(4), 389–395. As noted above, other studies also suggest that some women who have elected to receive VUS are able to use this information in a meaningful way.

31 Cf. McGillivray et al., op. cit. note 31.

32 For discussion of a clinical situation in which analogous information is justifiably withheld, see Deans, Z., Clarke, A. J., & Newson, A. J. (2015). For your interest? The ethical acceptability of using non‐invasive prenatal testing ‘purely for information’. *Bioethics, 29*(1), 19–25.

33 van der Steen et al., op. cit. note 5; Millo, T., Douiev, L., Popper, D., & Shkedi‐Rafid, S. (2020). Personalized prenatal genomic testing: Couples' experience with choice regarding uncertain and adult‐onset findings from chromosomal‐microarray‐analysis. *Prenatal Diagnosis, 41*(3), 376–383. https://doi.org/10.1002/pd.5856

34 Halliday, J. L., Muller, C., Charles, T., Norris, F., Kennedy, J., Lewis, S., … Amor, D. J. (2018). Offering pregnant women different levels of genetic information from prenatal chromosome microarray: A prospective study. *European Journal of Human Genetics, 26*(4), 485–494.

35 Ibid.

36 McGillivray et al., op. cit. note 31; and see, for example, Rosenfeld et al., op. cit. note 6; Stark et al., op. cit. note 6.

37 McGillivray et al., op. cit. note 31, p. 393.

38 Ibid: 389.

39 Cf. Walker, T. (2013). Respecting autonomy without disclosing information. *Bioethics, 27*(7), 388–394.

40 See Manson, N. C., & O'Neill, O. (2007). *Rethinking informed consent in bioethics* (pp. 18–22). Cambridge, U.K.: Cambridge University Press; Dive, L., & Newson, A. J. (2018). Reconceptualizing autonomy for bioethics. *Kennedy Institute of Ethics Journal, 28*(2), 171–203; Pugh, J. (2020). *Autonomy, rationality, and contemporary bioethics* (pp. 155–163). Oxford, U.K.: Oxford University Press.

41 We understand ‘appropriate’ responsiveness to reasons as the ability to perceive whether certain actions are consistent, or inconsistent, with the values that are relevant to the decision to act. It may be that impersonal values, rather than simply the personal values of the agent, are relevant to decision‐making that conforms to norms of deliberative reflection and therefore to genuinely autonomous decision‐making. We remain neutral on this.

42 Manson and O'Neill (op. cit. note 41, pp. 69–72) argue that insofar as respect for a person's autonomy requires *ensuring* that their decisions conform to norms of deliberative reflection, then this requirement is too demanding in the context of informed consent, and thus we ought to rethink whether respect for autonomy is an appropriate ideal in this context. Against this, Pugh (op. cit. note 41, pp. 162–163) argues that we can retain respect for autonomy as an ideal if we understand our doing so to require *facilitating* choices that conform to norms of deliberative reflection—for how can we seek to *ensure* that a person's choices conform to norms of deliberative reflection and still think of them as *autonomous* decision‐makers? We follow Pugh here.

43 It could be objected here that deliberation is often a process of seeking to find a justification for a decision made on grounds other than reasoned reflection. Yet, even so, to the extent that one supposes such deliberation to *justify* one's decisions, then one appeals to norms of deliberative reflection as a *further* ground for making the decision, and one's decisions can be either endorsed or criticized depending upon how closely they conform to, or depart from, these norms.

44 van der Steen, S. L., Bunnik, E. M., Polak, M. G., Diderich, K. E. M., Verhagen‐Visser, J., Govaerts, L. C. P., … Riedijk, S. R. (2018). Choosing between higher and lower resolution microarrays: Do pregnant women have sufficient knowledge to make informed choices consistent with their attitude? *Journal of Genetic Counselling, 27*, 85–94.

45 Here, we note conflicting literature regarding the quality of the choices or decisions that pregnant women actually make regarding VUS: Baker et al., op. cit. note 16; cf. van der Steen et al., op. cit. note 45.

46 McGillivray et al., op. cit. note 31, p. 393.

47 See, for example, de Jong et al., op. cit. note 6; Kater‐Kuipers, A., de Beaufort, I. D., Galjaard, R. H., & Bunnik, E. M. (2018). Ethics of routine: A critical analysis of the concept of ‘routinization’ in prenatal screening. *Journal of Medical Ethics, 44*(9), 626–631; Bennett, R. (2001). Antenatal genetic testing and the right to remain in ignorance. *Theoretical Medicine and Bioethics, 22*(5), 461–471.

48 See de Jong et al., op. cit. note 6; Kater‐Kuipers et al., op. cit. note 48; Deans Z., & Newson A. J. (2011). Should non‐invasiveness change informed consent procedures for prenatal diagnosis? *Health Care Analysis, 19*(2), 122–132; Newson, A. J. (2008). Ethical aspects arising from non‐invasive fetal diagnosis. *Seminars in Fetal & Neonatal Medicine, 13*(2), 103–108; Suter, S. M. (2002). The routinization of prenatal testing. *American Journal of Law & Medicine, 28*(2–3), 233–270.

49 Kater‐Kuipers et al., op. cit. note 48, p. 628.

50 Indeed, we believe that the two‐tier model developed by Halliday et al. (op. cit. note 35) is a best‐practice example of such an offer.

51 See, for example, Kater‐Kuipers et al., op. cit. note 48.

52 McGillivray et al., op. cit. note 31, p. 393.

53 Recall that on all the models we propose, general information regarding potential VUS is provided during pre‐test counselling; it is access to specific information concerning actual VUS in post‐test counselling that is at issue.

54 Hofmann op. cit. note 28. On the related question of what should be said to constitute a ‘prenatal result’, see Shkedi‐Rafid, S., Horton, R., & Lucassen, A. (2020). What is the meaning of a ‘genomic result’ in the context of pregnancy? *European Journal of Human Genetics, 27*(2), 183–187. https://doi.org/10.1038/s41431-020-00722-8

55 We do not imagine that these are the only reasons one could give to justify preferring a *Request Model* over a *Routine Offer Model*. Rather, we give initial presentations of these two reasons on account of their being both independently interesting and consistent with respect for reproductive autonomy.

56 Kolers, A. (2020). What does solidarity do for bioethics? *Journal of Medical Ethics, 47*(2), 122–128. https://doi.org/10.1136/medethics-2019-106040; Jennings, B., & Dawson, A. (2015). Solidarity in the moral imagination of bioethics. *Hastings Center Report, 45*(5), 31–38; Prainsack, B., & Buyx, A. (2012). Solidarity in contemporary bioethics—Towards a new approach. *Bioethics, 26*(7), 343–350.

57 Kolers op. cit. note 56, pp. 4–5; Jennings & Dawson, op. cit. note 56, pp. 33–35.

58 Hashiloni‐Dolev, Y., Nov‐Klaiman, T., & Raz, A. (2019). Pandora's pregnancy: NIPT, CMA, and genome sequencing: A new era for prenatal genetic testing. *Prenatal Diagnosis, 39*(10), 859–865.

59 Ibid: 859.

60 Ibid: 863. This point may be contentious in the genetic counselling profession. For instance, there is a growing literature regarding how to best support pregnant women to adapt to ambiguity and uncertainty inherent to contemporary prenatal testing: Werner‐Lin et al., op. cit. note 1; Richardson & Ormond, op. cit. note 12; Harding et al., op. cit. note 10. Others suggest that ambiguous information could be used in ‘conjoint knowledge production’ between health professionals and pregnant women: Lou et al., op. cit. note 8. However, it is also important to note that this standard of care will certainly not be the reality for many pregnant women, particularly if CMA becomes routine in all pregnancies. Women may have their test arranged by a health professional with no genetics training, and/or may not have access to adequate pre‐ and post‐test counselling.

61 Werner‐Lin et al., op. cit. note 1, p. S62.

62 Hashiloni‐Dolev et al., op. cit. note 58, p. 860.

63 Werner‐Lin et al., op. cit. note 1.

64 Ibid; Baker et al., op. cit. note 16.

65 Werner‐Lin et al., op. cit. note 1, pp. S63–S64.

66 See Sepielli, A. (2018). Decision making under moral uncertainty. In A. Zimmerman, K. Jones & M. Timmons (Eds.), *The Routledge handbook of moral epistemology* (pp. 508–521). Abingdon, U.K.: Routledge.

67 Cf. Lockhart, T. (2000). *Moral uncertainty and its consequences*. Oxford, U.K.: Oxford University Press (see especially pp. 3–21).

68 We recognize that to assume that moral understanding improves over time is to adopt a substantive meta‐ethical position. Nevertheless, insofar as we are engaged in a practical exercise in this paper, this is a reasonable working assumption to hold.

69 The ‘precautionary principle’ is controversial. Still, on the merits of principled precaution, see Hughes, J. (2006). How not to criticize the precautionary principle. *Journal of Medicine and Philosophy, 31*(5), 447–464.

70 We thank an anonymous reviewer for pushing us to clarify this point.

